# Identification and Characterization of *WRKY41*, a Gene Conferring Resistance to Powdery Mildew in Wild Tomato (*Solanum habrochaites*) LA1777

**DOI:** 10.3390/ijms23031267

**Published:** 2022-01-23

**Authors:** Qinggui Lian, Xinyi He, Bingbing Zhang, Yang Wang, Qing Ma

**Affiliations:** College of Plant Protection, Northwest A&F University, Xianyang 712100, China; lqg@nwafu.edu.cn (Q.L.); hexinyi@nwafu.edu.cn (X.H.); zhang_ure@163.com (B.Z.)

**Keywords:** tomato powdery mildew, *Oidium neolycopersici*, *WRKYs*, resistance

## Abstract

WRKYs, a large family of transcription factors, are involved in plant response to biotic and abiotic stresses, but the role of them in tomato resistance to *Oidium neolycopersici* is still unclear. In this study, we evaluate the role of *WRKYs* in powdery mildew-resistant wild tomato (*Solanum habrochaites*) LA1777 defense against *O. neolycopersici* strain lz (*On*-lz) using a combination of omics, classical plant pathology- and cell biology-based approaches. A total of 27 *WRKYs*, belonging to group I, II, and III, were identified as differentially expressed genes in LA1777 against *On*-lz. It was found that expression of *ShWRKY41* was increased after *Pseudomonas syringae* pv. tomato (*Pst*) DC3000, *On*-lz and *Botrytis* *cinerea* B05 inoculation or ethylene precursor 1-aminocyclopropane-1-carboxylic acid (ACC) treatment. GUS staining of *ShWRKY41* promoter indicated that the expression of *ShWRKY41* could be induced by SA and ethylene. Furthermore, *ShWRKY41* gene silencing reduced the resistance to *On*-lz infection by decreasing the generation of H_2_O_2_ and HR in LA1777 seedlings. Overall, our research suggests that *ShWRKY41* plays a positive role in defense activation and host resistance to *O. neolycopersici* in wild tomato (*S. habrochaites*) LA1777.

## 1. Introduction

Tomato powdery mildew, mainly caused by the obligate biotrophic fungus *Oidium neolycopersici* [1], is a fungal disease that commonly occurs in tomato leaves, characterized by chlorosis, white plaque and occasional necrosis [2,3]. Generally, this disease causes up to 50% yield (fruit) losses in heavily infected fields. Lack of resistant cultivars makes tomato powdery mildew become the limiting factors in tomato production [4]. Fortunately, *S. habrochaites*, a wild tomato germplasm resource and closely related to tomato cultivar *S. lycopersicum*, has a high resistance to the powdery mildew [5]. After *O. neolycopersici* infection, defense responses, including increased production of reactive oxygen species (ROS) and hypersensitive response (HR) are activated in resistant wild tomato S. *habrochaites* or *S. hirsutum*, but not in the susceptible *S. lycopersicum* cultivar tomato [5,6,7]. In wild tomato, some alleles have been identified as resistance genes, including *Ol-1*, *ol-2*, *Ol-3*, *Ol-4*, *Ol-5*, *Ol-6*, etc., the mechanisms of which are similar to the *MLO* gene in barley [5,6,7,8]. And *ShARPC3*, encoding a subunit protein of the actin-related protein 2/3 complex, is involved in resistance to *O. neolycopersici* in wild tomato *S. habrochaites* LA1777 by regulating the induction of hypersensitive cell death and the generation of reactive oxygen [9]. Also, *ShORR-1* (*S. habrochaites Oidium Resistance Required-1*), identified from *S. habrochaites* G1.1560, enhances host resistance to *O. neolycopersici* by effecting the extensive hydrogen peroxide accumulation and formation of abnormal haustoria [10], but the role of *WRKYs* in wild tomato *S. habrochaites* LA1777-*O. neolycopersici* interaction is still unclear.

WRKYs are a large family of transcription factors (TF) that seem to have originated in early eukaryotes [11]. As transcription factors, WRKYs are involved in plant responses to biotic and abiotic stresses [12,13,14,15,16] by altering the expression of other genes. SWEET POTATO FACTOR1 (SPF1) is the first WRKY protein, which is identified from sweet potato in 1994 and binds to the SP8a (ACTGTGTA) and SP8b (TACTATT) sequences of target genes [17]. Usually, WRKY proteins contain WRKY domain, which is a DNA-binding region of approximately 60 amino acids in length and comprises the conserved sequence motif WRKY adjacent to a novel zinc-finger motif. To date, lots of *WRKY* genes have been identified from *Arabidopsis* [18], tomato [19,20], rice [21], wheat [22], tobacco [23], etc. The WRKYs are divided into three groups, based on the number of WRKY domains and the types of zinc-finger-like motifs [24].

Functional redundancy among WRKY family members is a hindrance to linking specific WRKY TFs to plant defenses [25]. Some WRKYs directly/indirectly interact with pathogen-associated molecular patterns (PAMPs) or effector proteins to activate or suppress PAMP-triggered immunity (PTI) and/or effector-triggered immunity (ETI) [26], which are two tiers in plant defense responses against invading pathogens [27,28]. In *Arabidopsis*, *WRKY18*, *WRKY40*, and *WRKY60* redundantly and cooperatively function as negative regulators of PTI to *Pseudomonas syringae* [29] and *Hv*WRKY2 (*Hv, Hordeum vulgare*) also functions as a negative regulator of PTI to powdery mildew fungus *Golovinomyces orontii* by interacting with barley mildew A (MLA) R protein [30]. In rice, *OsWRKY62* gene functions as a negative regulator of innate immunity and serves as a critical mediator of both basal and race-specific defense responses [31]. Meanwhile, some WRKYs work as a positive regulator to participate in plant resistance against phytopathogens. In rice (*Oryza sativa*), *OsWRKY13* [32], *OsWRKY45* [33], *OsWRKY53* [34], and *OsWRKY67* [35] defend against invasion of different pathogens by activating or suppressing the expression of diverse downstream targeting genes to finally moderate plant physiological performance, alter plant phytohormone balance, or modify plant transcriptomic network.

In tomato, a total of 83 *WRKYs* are identified [20], some of which function as positive regulators of plant responses to biotic stresses, e.g., *SlWRKY31* [36], *SlWRKY33* [37], *SlWRKY39* [38]. Lots of *WRKY* genes have been identified from tomato-pathogens interaction: *SlWRKY45* enhancing tomato susceptibility to the root-knot nematode *Meloidogyne javanica* [39], *SlWRKY39* enhancing resistance to *P. syringae* [38], and *SlWRKY33* enhancing resistance to hemi-biotrophic oomycete *Phytophthora infestans*, etc. However, there is a little knowledge about the function of WRKYs in wild tomato *S. habrochaites* LA1777 defense against *O. neolycopersici*. An understanding the role of WRKYs between wild tomato LA1777 and *O. neolycopersici* will provide a foundation for tomato anti-disease and breeding. Here, we reveal the expression patterns of *WRKY* genes in wild tomato LA1777, a powdery mildew resistance tomato, under *O. neolycopersici* strain lz infection. And further studies find that *ShWRKY41*, induced by ethylene, is positively related to wild tomato LA1777 defense against *On*-lz.

## 2. Results

### 2.1. A Total of 27 WRKYs Are Differentially Expressed in LA1777 under On-lz Infection

**Figure 1:**
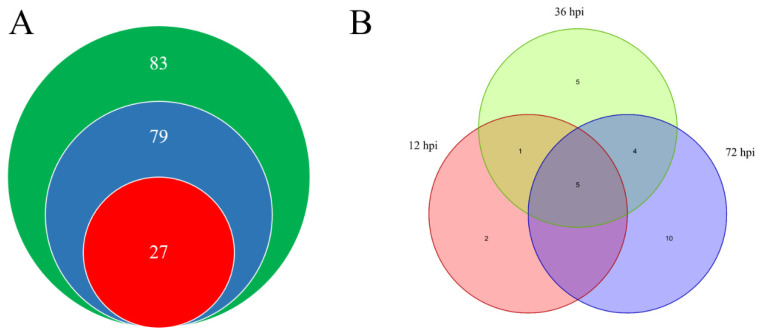
The differentially expressed *WRKY* genes in LA1777 under *On*-lz infection. In figure (**A**), red color represents the differentially expressed *WRKY* genes, and blue color represents the *WRKY* transcripts in LA1777 under *On*-lz infection and green color represents the *WRKY* genes in *S. lycopersicum* genome. (**B**) The distribution of differentially expressed *WRKY* genes at different time points.

A total of 83 *WRKYs* were identified in *S. lycopersicum* in previous research [19,20]. In this study, 69 transcripts were mapped to *WRKY* genes (Supplementary Table S1). With the criteria of padj (the adjusted *p* value) < 0.05 and |log2FoldChange| > 1, a total of 27 *WRKYs* were identified as differentially expressed genes (DEGs) (Figure 1A) in wild tomato *S. habrochaites* LA1777 under *On-lz* infection. We found that eight *WRKYs* were differentially expressed in LA1777 at 12 hpi, and 15 *WRKYs* were differentially expressed in LA1777 at 36 hpi, and 19 *WRKYs* were differentially expressed in LA1777 at 72 hpi (Figure 1B). With progress of infection, the number of differentially expressed *WRKY* genes increased.

### 2.2. Categorization and Function Analysis of Differentially Expressed WRKYs in Wild Tomato LA1777 under On-lz Infection

According to a former study [40], the 27 differentially expressed *WRKY* genes were classified as I, II-a, II-c, II-d, II-e, and III group (Table 1) based on the sequence variation among WRKY proteins.

At 12 hpi, eight *WRKYs* were identified as DEGs, and the most were up-regulated except *ShWRKY51*. *ShWRKY44* and *ShWRKY1* that formed group I, and the others (*ShWRKY40*, *ShWRKY51*, *ShWRKY IId-1*, *ShWRKY22-like*, *ShWRKY29*, and *ShWRKY41*) belonged to groups II-a, II -c, II -d, II –e, II –e, and III, respectively. At 36 hpi, the number of *WRKY* DEGs was increased to 15. Like to 12 hpi, only *ShWRKY3*, belonging to group I, was a down-regulated DEG, and the others were up-regulated DEGs. At 72 hpi, the number of *WRKY* DEGs was up to 19. Meanwhile, we found three down-regulated DEGs: *ShWRKY14* (group I), *ShWRKY75* (group II -c), and *ShWRKY21* (group II -d).

**Figure 2:**
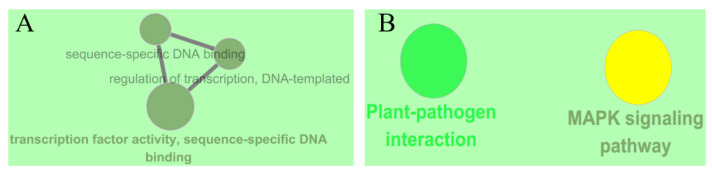
The function analysis of differentially expressed *WRKYs* in wild tomato *S. habrochaites* LA1777 under *On*-lz infection. (**A**) The result of GO enrichment on molecular function term, and (**B**) The result of KEGG enrichment. The GO and KEGG enrichment were both analyzed in Cytoscope using ClueGO app.

To know the function of the differentially expressed *WRKYs*, all of those *WRKYs* were analyzed using ClueGO in Cytoscope. As Figure 2A shown, like other *WRKYs*, the molecular function of wild tomato *ShWRKYs* was transcription factor activity, sequence-specific DNA binding. And KEGG analysis (Figure 2B) demonstrated that differentially expressed *WRKYs* were significantly enriched to MAPK signal and plant-pathogen interaction pathway.

**Figure 3:**
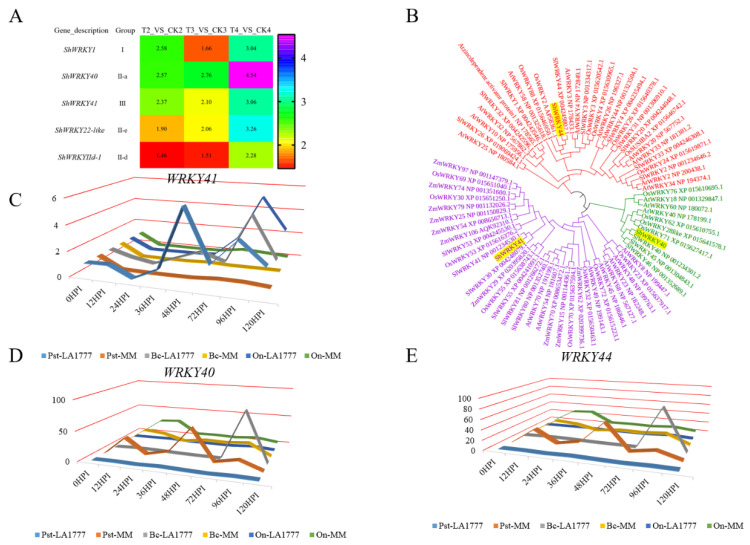
The screening and identification of *WRKY41*. (**A**) The co-expressed *WRKY* genes among the RNA-seq analysis. (**B**) The result of phylogenetic analysis of WRKY40, WRKY41 and WRKY44 using NJ method with JTT model. (**C**–**E**) The result of *WRKY40*, *WRKY41* and *WRKY44* relative expression levels in wild tomato LA1777 or cultivar tomato MM under *Pst* DC3000, *On*-lz and *B. cinerea* infection using qRT-PCR.

Among those DEGs, five *WRKYs* (*ShWRKY1*, *ShWRKY40*, *ShWRKY41*, *ShWRKY22-like*, and *ShWRKY IId-1*) were identified as simultaneous genes at three time points, and all of them were up-regulated (Figure 3A), and *ShWRKY44* had highest expression level at 12 and 36 hpi (4.99 and 5.73, respectively), in wild tomato LA1777 under *On*-lz infection. Group I *ShWRKY44*, group II *ShWRKY40,* and group III *ShWRKY41* were selected as a representative of each of three groups for a further analysis. The result of phylogenetic analysis (Figure 3B), based on the amino acid sequence, showed that ShWRKY40, ShWRKY41, and ShWRKY44 were classified to different cluster. ShWRKY40, SlWRKY40/45, OsWRKY71, AtWRKY40, etc. formed one cluster, and ShWRKY41, SlWRKY41, OsWRKY53, etc. formed one cluster, and ShWRKY44, SlWRKY44, OsWRKY88, etc. formed one cluster. The result of categorization and phylogenetic analysis indicated that ShWRKY40, ShWRKY41, and ShWRKY44 belonged to different WRKY groups.

The expression levels of *ShWRKY40*, *ShWRKY41*, and *ShWRKY44* under biotrophic pathogens (*On*-lz and *Pst* DC3000) and nectrophic pathogen *B. cinerea* B05 were analyzed. The results (Figure 3C–E) showed that *ShWRKY41*, different from *ShWRKY40* and *ShWRKY44*, had higher expression levels in wild tomato LA1777, but lower expression levels in cultivar tomato MM, under *On*-lz, *Pst* DC3000, and *B. cinerea* B05 infections.

Based on the above result, we found that *ShWRKY41*, a member of group III, had high expression levels in wild tomato LA1777 under biotrophic and nectrophic pathogens. So, the molecular function of *ShWRKY41* was further studied.

### 2.3. The Molecular Function Analysis of ShWRKY41

The amino acid sequence of ShWRKY41 was used for STRING analysis based on the *Solanum lycopersicum* database. We found that ShWRKY41 putatively interacted with pathogen-related protein, PR1 protein and MLO-like protein 6 (Figure S1A), which indicated ShWRKY41 was involved in disease resistance in wild tomato LA1777. While, the accumulation of mRNAs, coding the putative interacted proteins, was analyzed using RNA-seq data. Our data showed that they were mainly down-regulated genes (Figure S1C), but the expression of them were not found to be statistically significant at the level padj < 0.05 except *PR1* at 72 hpi (marked with T4). The result of Pfam analysis (Figure S1B) showed that ShWRKY41 contained one WRKY domain (137–197 aa).

**Figure 4:**
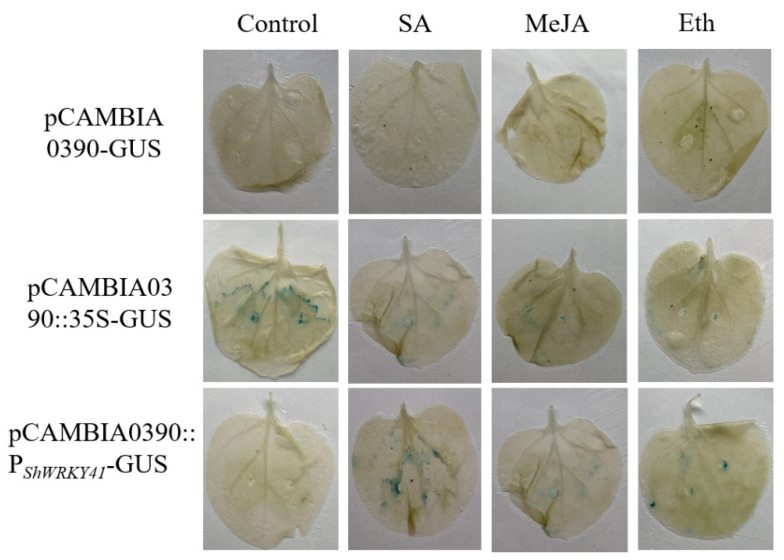
The GUS analysis of wild tomato *ShWRKY41* promoter. Histochemical GUS assay was performed in the transient expression *N. benthamiana*, which were cultivated in a 22 °C chamber with 16 h light/8 h dark cycle for 2 days after the treatment with 100 mM MeJA, 10 mM SA, 0.5 mM ACC (1-aminocyclopropanecarboxylic acid, the ethylene precursor) (Sigma, Shanghai, China), and water (Control), respectively.

To know more about the expression activity of *ShWRKY41*, a promoter analysis was analyzed by PlantCARE and Softberry. The *cis*-acting elements, involved in the SA, MeJA, or Eth responsiveness, were not found in the promoter of *ShWRKY41*. GUS assay was employed to reveal promoter responsiveness, and as shown in Figure 4, after SA or Eth treatment, GUS activity was highly induced in pCAMBIA0390:: P*_ShWRKY41_*-GUS, which indicated that the promoter fusions were responsive to SA, MeJA, and ethylene precursor ACC treatment.

**Figure 5:**
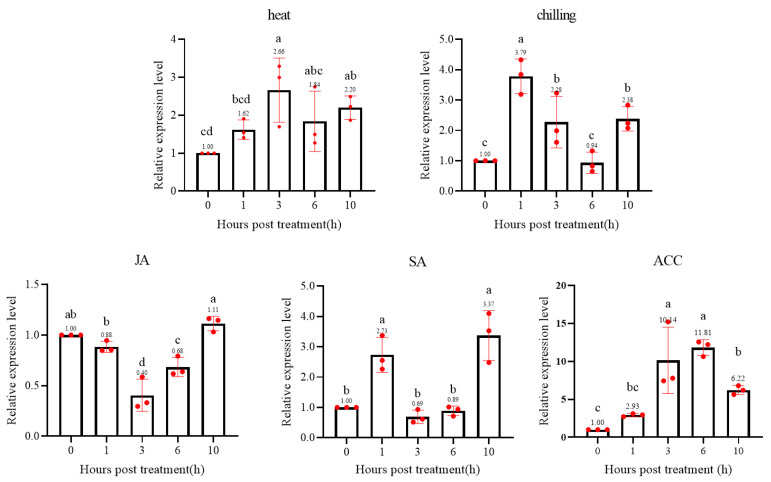
The expression of *ShWRKY41* in wild tomato *S. habrochaites* LA1777 under abiotic stresses. ‘heat’ means a 40 °C high temperature stress, and ‘chilling’ represents a 8 °C low temperature stress. ‘JA’, ‘SA’, and ‘ACC’ mean plant hormones stresses. The different lowercase letters (e.g., a, b, c, etc.) represent the significance at *p* = 0.05 level, and if there are one or more same letters between different groups (e.g., ab, bc, abc, etc.), it means there is a difference between those groups but not significant.

**Figure 6:**
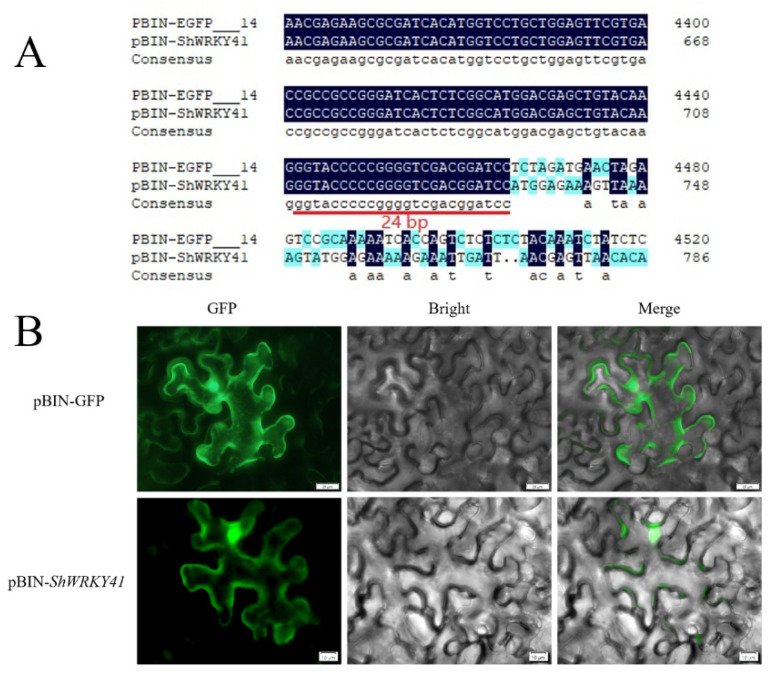
Sh*WRKY41* located in plasma membrane and nucleus. (**A**) The sequence analysis of pBIN-*ShWRKY41* vector by Sanger sequencing. The 24 bp gap means that the GFP coding sequence and CDS of *ShWRKY41* did not exist frameshift mutation. (**B**) Images were collected by confocal microscopy at 24 h post pBIN-*ShWRKY41* inoculation. Green fluorescent protein (GFP) signal was visualized by confocal microscopy.

Then, the expression pattern was analyzed using qRT-PCR. As the Figure 5 shown, the expression levels of wild tomato *ShWRKY41* was increased after heat (40 °C), chilling (8 °C), SA, and ACC treatments, but decreased after JA treatment (Figure 6). The expression patterns of wild tomato *ShWRKY41* after heat and ACC treatments were similar, both of which were continuously increased. Meanwhile, the expression patterns of *ShWRKY41* after chilling and SA treatments were similar, both of which had a high expression level at 1 and 10 hpi. While, the expression of wild tomato *ShWRKY41* was reduced after JA treatment, down to 0.40 at 3 hpi, and increased back up to 1.11 at 10 hpi.

By in silico analysis, it was predicted that wild tomato *ShWRKY41* was primarily located in the plant plasma membrane and nucleus. To validate this prediction, a 1010 bp fragment of *ShWRKY41* was cloned into the binary expression vector pBIN-*EGFP*. After validation using Sanger sequencing (Figure 6A), the recombinant vector was transformed into *A. tumefaciens* strain GV3101 and transiently expressed in *N. benthamiana*. As shown in Figure 6B, at 24 h post-co-infiltration, pBIN-*ShWRKY41* expressed in plasma membrane and nucleus in tobacco cell, which was similar with the control infiltration (i.e., pBIN-*EGFP*). This result confirmed the prediction of localization pattern.

### 2.4. ShWRKY41 Silencing Results in Host Susceptibility to On-lz in Wild Tomato LA1777 Seedlings

**Figure 7:**
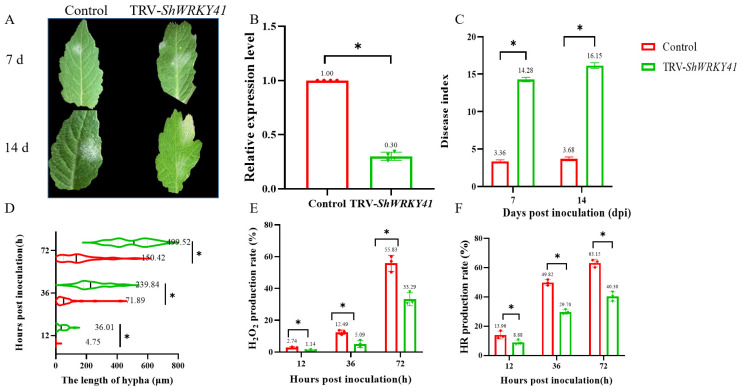

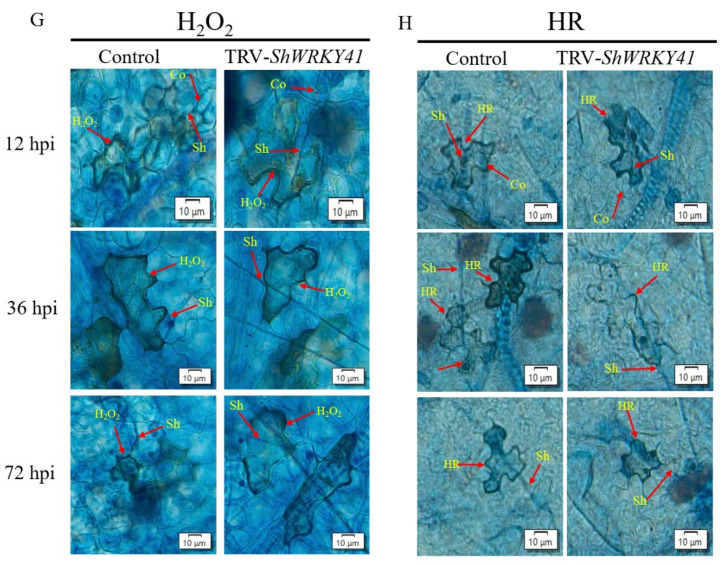
The silence of *ShWRKY41* reduces the resistance of wild tomato LA1777 against *On*-lz. In the figure, the red legend represents the data from tomato LA1777 seedlings and the green legend represents the data from *ShWRKY41*-silenced LA1777 seedlings. (**A**) Phenotype of TRV (control) or TRV-*ShWRKY41*-silenced seedings under *On*-lz infection at 7&14-day-post-inoculation. (**B**) The efficiency of *ShWRKY41* silencing in LA1777 seedlings. (**C**) The quantification of disease TRV-*ShWRKY41*-silenced or control LA1777 seedlings at 7- and 14-day-post-inoculation with *On*-lz. (**D**) The statistics of *O. neolycopersici* hypha on *ShWRKY41*-silenced or control LA1777 seedlings. (**E**) The H_2_O_2_ production rate in *ShWRKY41*-silenced or control LA1777 seedlings under *On*-lz infection. (**F**) The HR induction rate in *ShWRKY41* silenced or control LA1777 seedlings under *On*-lz infection. The asterisk indicates statistically significant differences at level α = 0.05 with Student’s *t*-test between control and TRV-*ShWRKY41*-silenced wild tomato LA1777 seedlings. (**G**,**H**) The microscopic detection of H_2_O_2_ and HR accumulations at interaction sites of *O. neolycopersici* with control and silenced *ShWRKY41*, respectively. Co, conidium; Sh, secondary hyphae. Bar, 10 mm.

To evaluate the role of *ShWRKY41* during the interaction between wild tomato *S. habrochaites* LA1777 seedlings and *On*-lz, tobacco rattle virus-based virus-induced gene silencing (TRV-VIGS) was used to silence *ShWRKY41* in wild tomato LA1777 seedlings. TRV1, TRV2, TRV2-*SlPDS* (*phytoene desaturase*), and TRV2-*ShWRKY41* plasmids were transformed into *A. tumefaciens* strain GV3101, respectively, to silence the target genes in wild tomato LA1777 seedlings. To evaluate the efficiency of the TRV-based approach, TRV2-*SlPDS* was monitored for the induction of photobleaching. Five weeks after inoculation, a photobleaching phenotype was observed, indicating the success of technical efficiency of TRV-VIGS-mediated gene silencing. The result of qRT-PCR showed that the expression of *ShWRKY41* was 0.30 in the TRV2-*ShWRKY41* silenced seedlings, which indicated a high degree of silencing efficiency (Figure 7B). In parallel to the analysis of phenotypes, all TRV-VIGS seedlings were inoculated with *On*-lz and the infection phenotypes were recorded. Compared with control plants, plants carrying TRV2-*ShWRKY41* appeared obvious powdery mildew disease lesions (Figure 7A). The disease index values of *ShWRKY41* silenced seedlings (7 dpi: 14.28 and 14 dpi: 16.15) were significantly higher than control seedlings (7 dpi: 3.36 and 14 dpi: 3.68) (Figure 7C).

Histological observations showed that the growth of *On*-lz conidia on the *ShWRKY41* silenced seedlings was faster than that on the control seedlings. At 12 hpi, the hypha length showed that the average of hypha length was 36.01 μm on the *ShWRKY41* silenced seedlings, which was significantly higher than 4.75 μm on the control seedlings. At 36 hpi, the average of hypha length on the *ShWRKY41* silenced seedlings was 239.84 μm, while the average of hypha length on the control seedlings was only 71.89 μm. At 72 hpi, the length of *On*-lz hyphae on the control seedlings was 150.42 μm, significantly lower than 499.52 μm on the *ShWRKY41* silenced seedlings (Figure 7D).

To further demonstrate the function of *ShWRKY41* in wild tomato LA1777 resistance to *On*-lz infection, we evaluated the induction of early defense signaling processes, such as the accumulation of H_2_O_2_ and the induction of hypersensitive response (HR). In the case of H_2_O_2_ response signaling (Figure 7E,G), at 12 hpi, 2.74% of the infection sites from control seedlings produced H_2_O_2_, which was higher than that of *ShWRKY41*-silenced seedlings. At 36 hpi, in control seedlings, the production of H_2_O_2_ was observed to be 12.49%, which was significantly higher than that of *ShWRKY41*-silenced seedlings (*p* value = 0.05). At 72 hpi, control seedlings produced more H_2_O_2_ (55.83%) than *ShWRKY41*-silenced seedlings (33.29%). A similar trend was observed for the induction of the HR, which revealed that *ShWRKY41* silencing reduced the induction of HR (Figure 7F,H). At 12 hpi, the rate of HR induction was 13.96% in control seedlings, which was higher than 8.88% in *ShWRKY41*-silenced seedlings. At 36 hpi, in *ShWRKY41*-silenced seedlings, the rate of HR induction was 49.82%, which was significantly higher than 29.70% in *ShWRKY41*-silenced seedlings. At 72 hpi, control seedlings were induced more HR (63.15%) than *ShWRKY41*-silenced seedlings (40.30%).

**Figure 8:**
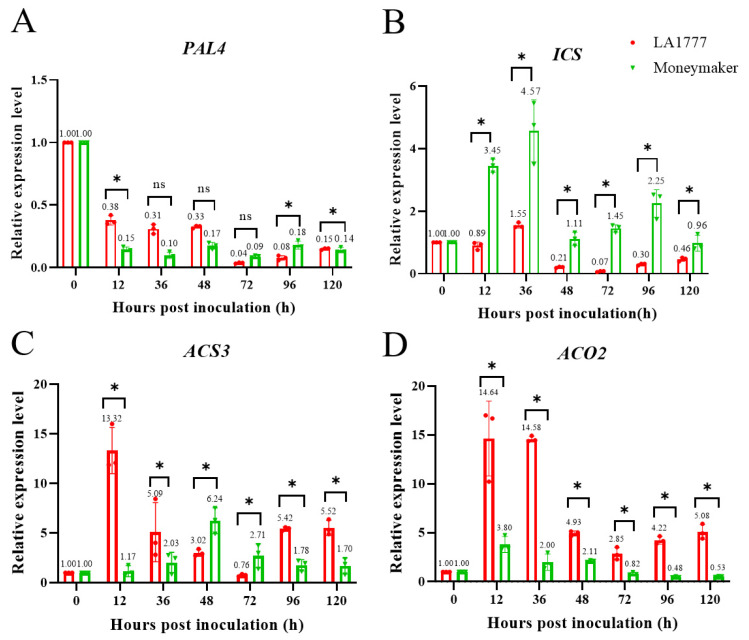
The expression of *PAL4* and *ICS* are suppressed, but the expression of *ACS3* and *ACO2* are activated in wild tomato LA1777 under *On*-lz infection. In the figure, the red legend represents the data from tomato LA1777 seedlings and the green legend represents the data from cultivar tomato MM. (**A**) The expression level of *PAL4*. (**B**) The expression level of *ICS*. (**C**) The expression level of *ACS3.* (**D**) The expression level of *ACO2*. The asterisk indicates statistically significant differences and ‘ns’ indicates no statistically significant differences at level α = 0.05 with Student’s *t*-test between wild tomato LA1777 and cultivar tomato MM seedlings.

Based on the above result, we found that *ShWRKY41*, which could be induced by SA and Eth, was involved in wild tomato LA1777 defense against *On*-lz. To know which plant hormone was involved in LA1777 against *On*-lz, genes, *PAL4* and *ICS*, which were related to SA biosynthesis, *ACS3* and *ACO2*, which were related to Eth biosynthesis, were analyzed using qRT-PCR. We found that the expression levels of *PAL4* and *ICS* were decreased in wild tomato LA1777 under *On*-lz infection (Figure 8A,B), while the expression levels of *ACS3* and *ACO2* were increased in wild tomato LA1777 under *On*-lz infection (Figure 8C,D). This result indicated that Eth was involved in wild tomato LA1777 defense against *On*-lz, instead of SA. So, the gene *ShWRKY41* was induced by Eth in the process of wild tomato LA1777 against *On*-lz.

## 3. Discussion

*Oidium neolycopersici*, a biotrophic fungus, is the main pathogen responsible for tomato powdery mildew [1]. A lack of resistant cultivars makes tomato powdery mildew become the limiting factor in tomato production [4]. Fortunately, previous research has led to the discovery of wild germplasm resources, e.g., *Solanum habrochaites,* which have resistance to *O. neolycopersici* infection [5], and our former studies found that *ShARP3* and *ShNPSN11* are involved in resistance to powdery mildew in wild tomato *S. habrochaites* LA1777 [14,20]. Nevertheless, what the function of *WRKYs* in the crosstalk between resistant wild tomato LA1777 and *O. neolycopersici* is unclear. WRKYs, as a large family of transcription factors, functionally connect and form a transcriptional network, then involves in plant immune responses through the regulation of transcriptional reprogramming [18,25,26]. In this study, we used next generation sequencing (NGS) technology to illuminate the possible role of *WRKYs* in LA1777 against *O. neolycopersici* strain lz (*On*-lz). A total of 27 *WRKYs* were identified as differentially expressed genes with padj ≤ 0.05 and |fold-change| ≥1. These genes were mainly enriched to the transcription factor activity, sequence-specific DNA binding at GO term of molecular function and enriched to MAPK signal pathway and plant-pathogen interaction in KEGG analysis.

WRKYs are categorized into group I, II, and III based on the number of WRKY domains and the type of zinc-finger-like motif [24]. In this study, the differentially expressed genes were distributed into tomato *WRKY* group I, II and III (Table 1). Among them, 18 differentially expressed *WRKYs* belonged to group II, accounting for 66.67%, and six differentially expressed *WRKYs* belonged to group I, accounting for 22.22%, and there are only three differentially expressed genes in group III, accounting for 11.11%. We also found three down-regulated expressed genes, *WRKY21*, *WRKY51*, and *WRKY75*, belonged to II group, and two down-regulated expressed genes, *WRKY3* and *WRKY14*, belonged to I group.

Some studies have revealed that *WRKY75*, a member of group II, is involved in response to necrotrophic pathogens infection and abiotic stresses [41,42], and *WRKY3*, a member of group I, is involved in resistance to *Phytophthora infestans* infection and salt stress tolerance [43,44], in tomato. In *Arabidopsis*, the mutation of *AtWRKY18*, *AtWRKY40* and *AtWRKY60*, members of the WRKY II subfamily, result in increased resistance to *P. syringae* and increased susceptibility to *B. cinerea* through inducing the expression of SA–regulated *PR1* and JA-regulated *PDF1.2* [29]. The result above suggest that members of this subfamily may work as negative regulators in plant defense biotrophic pathogens. While the other 22 *WRKYs* had high expression levels, five of them (Figure 3A), including *WRKY1*, *WRKY40*, *WRKY41*, *WRKY22-like*, and *WRKYIId-1*, always had high expression levels at 12, 36, and 72 hpi in wild tomato LA1777 under *On*-lz infection conditions. Of them, *WRKY1* had been identified as a positive regulator in tomato *P. infestans* interaction [45,46]. We also found that *WRKY44* had highest expression levels at 12 and 36 h after *On*-lz inoculation.

Next, three highly expressed *WRKYs—ShWRKY40*, *ShWRKY41*, and *ShWRKY44—*belonging to the group II, III, and I, respectively, were selected for further analysis. Based on the amino acid sequence, we found that ShWRKY40 had a high sequence similarity to SlWRKY40/45, rice OsWRKY71, and *Arabidopsis* AtWRKY40, and ShWRKY41 had a high sequence similarity to SlWRKY41, and OsWRKY53, and ShWRKY44 had a high sequence similarity to SlWRKY44, and OsWRKY88, indicating that ShWRKY40, ShWRKY41, and ShWRKY44 belonged to different WRKY groups (Figure 3B). And we also analyzed the expression levels of *ShWRKY40*, *ShWRKY41*, and *ShWRKY44* in wild tomato LA1777 and cultivar tomato MM under *Pst* DC 3000, *On*-lz, or *B. cinerea* B05 infection. The result indicated that the transcription of *ShWRKY41* was activated by those three pathogens, which revealed that *ShWRKY41* may be a positive regulator in wild tomato LA1777 response to different pathogens’ infection. *OsWRKY53*, having a high sequence similarity to *ShWRKY41*, has a functional diversity in rice, by activating or suppressing the expression of diverse downstream targeting genes to finally moderate plant physiological performance and interfere plant balance [34,47,48,49,50,51]. Phytohormones play a critical role in plant response to pathogens infection, and the biosynthesis of SA, Eth and JA is triggered after pathogens infection and then these hormones lead to activation of downstream signaling in cells producing them. Based on the previous reports, during powdery mildew resistance, the wild tomato resistance gene *Ol-1* and *Ol-qtls* need ET to generate cell death, and JA deficiency can compromise resistance mediated by *ol-2*, whereas *Ol-4*-mediated resistance depends on SA [5,6,7,8]. In this study, we found that expression of *ShWRKY41* was induced by plant hormones SA and Eth (Figure 5 and Figure 6) using GUS-promoter assay and qRT-PCR analysis, two major hormones involved in response to different stresses in plant [52].

Normally, to respond to biotic or abiotic stresses, plant hormones change the transcription level of related genes through transcription factors, e.g., WRKY transcription factors. Ectopic overexpression of *Capsicum annum* *WRKY27* confers resistance to *Ralstonia solanacearum* in *Nicotiana tabacum* through modulation of SA-, JA- and ET-mediated signaling pathways [53]. *MusaWRKY18*, from banana, P*_MusaWRKY18_*-β-D-glucuronidase (GUS) assay reveals that the expression of *MusaWRKY18* is strongly induced after application of abscisic acid, SA, methyl jasmonate, and ethephon, which indicates that plant hormones SA and Eth can induce the expression of *MusaWRKY18* [54]. *MiWRKY53*, identified from mulberry (*Morus indica* var. K2) and induced by SA, functions as a positive regulator of plant defense response through SA-mediated mechanisms [55]. In this study, to know which of them, SA and Eth, could induce the expression of *ShWRKY41*, and was involved in wild tomato LA1777 against *On*-lz, the expression level of genes, *PAL4* and *ICS*, coding the key enzymes of SA biosynthesis, *ACS3* and *ACO2*, coding the key enzymes of Eth biosynthesis, were analyzed using qRT-PCR. And the result (Figure 8) showed that *ACS3* and *ACO2* had high expression levels in wild tomato LA1777 under *On*-lz infection. Oppositely, *PAL4* and *ICS* had low expression levels. So, we inferred that Eth was involved in the resistance of wild tomato LA1777 against *On*-lz.

Some new studies reveal that Eth is involved in plant defense responses to biotrophic pathogens. During plant-biotrophic pathogen interactions, Eth has been demonstrated to be involved in *Arabidopsis* resistance to powdery mildew by feedback-attenuated RPW8.1-mediated cell death and disease resistance [56]. In contrast, Eth is needed in *Triticum urartu* resistance to *Blumeria graminis* f. sp. *tritici* (*Bgt*) infection because the expression of *TuACO3*, positively related to Eth biosynthesis, is induced by *Bgt* infection and accompanied by increased Eth content. *TuACO3*-silenced decreases Eth production and wheat resistance to *Bgt*, but both processes are enhanced in the overexpressed *TuACO3* wheat [57]. Similar to this, Eth biosynthesis-related genes had high expression levels in wild tomato *S. habrochaites* LA1777 under *On*-lz infection. Normally, ethylene could induce the generation of ROS by modulating the activity of NADPH oxidase-dependent H_2_O_2_ synthesis and a high ROS level could induce HR, in plant tissues [58,59]. Plants, resistance to biotrophic pathogens, e.g., *O. neolycopersici*, is closely related to the H_2_O_2_ accumulation and generation of HR triggered by the fungal haustoria [7]. In the study of tomato powdery mildew resistance, more HR and H_2_O_2_ are found in resistant tomatoes, including wild tomato *S. habrochaites* G1.1560 and *S. habrochaites* LA1777, compared to the susceptible *S. lycopersicum* MM tomato cultivar [9,10,60]. If the generation of HR and H_2_O_2_ is reduced, the resistance to biotrophic pathogens will be decreased [28,61]. Herein, we found that the silencing of *ShWRKY41* decreased the resistance to *On*-lz in wild tomato *S. habrochaites* LA1777 seedlings through reducing the production of H_2_O_2_ and HR (Figure 7), so the resistance, induced by Eth, was related to the ability of Eth-induced H_2_O_2_ generation. In conclusion, herein we reveal that *ShWRKY41* was involved in wild tomato *S. habrochaites* LA1777 defense against *On*-lz by regulating the generation of H_2_O_2_ and HR.

## 4. Materials and Methods

### 4.1. Plant Materials and Plant, Pathogen Growth, and Inoculation Experiments

*Oidium neolycopersici* strain lz (*On*-lz) was propagated and preserved according to the previously reported method [9,60]. Wild tomato *S. habrochaites* LA1777, obtained from the Tomato Genetics Resource Center (Department of Plant Sciences, University of California, Davis, CA, USA), and tomato Moneymaker (MM) (*S. lycopersicum*), were used in this research. Wild tomato *S. habrochaites* LA1777 is highly resistant to *On*-lz, while tomato Moneymaker is highly susceptible to *On*-lz. For germination and growth, tomato seeds were surface sterilized according to the method of the formers [60].

*Nicotiana benthamiana* seedlings were grown in a growth chamber at 20 °C under a 16 h light/8 h dark cycle with 60% relative humidity and a light intensity of 120-mmol photons m^−2^ s^−1^.

*Escherichia coli* strain DH5α was grown at 37 °C on Luria-Bertani (LB) medium containing antibiotics. *Agrobacterium tumefaciens* strain GV3101 harboring binary vector constructs was grown on antibiotic-containing LB media at 28 °C.

For pathogen inoculation assays, the conidia of *On*-lz were sprayed onto 8-day-old plants with a suspension of 10^5^ spores mL^−1^ according to the method of Zheng et al. [62]. Spore counts were quantified using a hemocytometer. Inoculated tomato seedlings were grown in environmentally controlled growth chambers under the same conditions as described above.

### 4.2. Sample Collection, Library Preparation and Transcriptome Sequencing

Fresh conidia were inoculated on wild tomato *S. habrochaites* LA1777 at 5-true-leaf stage by spraying spore suspension as described above. H_2_O was used as the control treatment. Based on the result of former study, the wild tomato LA1777 leaf tissues samples of 12 hpi, 36 hpi, and 72 hpi, collected from LA1777 under *On*-lz stress or control conditions, were used to RNA sequencing. Total RNA was extracted from the above samples by using the BioZol reagent (Bioer Technology, Hangzhou, China). RNAs were purified and used to construct sequencing libraries. The libraries were paired-end sequenced on the Illumina HiSeq platform. The library construction and sequencing were carried out at the Novogene Bioinformatics Technology Co., Ltd. (Beijing, China). And the raw data of RNA-seq was available at NCBI with the BioProject ID PRJNA769495.

### 4.3. Bioinformatics Analysis of WRKYs

Raw reads were processed by filtering out sequencing adapters, short-fragment reads, and other low-quality reads with FastQC v0.11.8 [63], Trimmomatic 0.38 [64] and FastUniq 1.1 [65]. Index of the reference genome *S. lycopersicum* Solyc2.50 (ftp://ftp.ncbi.nlm.nih.gov/genomes/all/GCF/000/188/115/GCF_000188115.3_SL2.50/ (accessed on 20 December 2017)) was built and paired-end clean reads were aligned to the reference genome by using Hisat2 v2.0.5 [66]. featureCounts v1.5.0-p3 [67] was used to count the reads numbers mapped to each gene, and then FPKM of each gene was calculated based on the length of the gene and reads count mapped to this gene. DESeq2 R package (1.16.1) [68] was used to identify the differentially expressed genes (DEGs). The resulting *p* values were adjusted using the Benjamini and Hochberg’s approach, called padj, for controlling the false discovery rate. The genes with padj ≤ 0.05 and |fold-change| ≥1 were identified as DEGs. And then the *WRKY* genes were obtained with perl scripts.

The predictions of expression-pattern and subcellular location were carried out in ProtParam (https://web.expasy.org/protparam/ (accessed on 21 November 2020)), Uniprot (https://www.uniprot.org/ (accessed on 21 November 2020)), and ProtComp (http://linux1.softberry.com/berry.phtml?topic=protcomppl&group=programs&subgroup=proloc (accessed on 21 November 2020)). The domains of ShWRKY proteins were checked with SMART database (http://smart.embl-heidelberg.de/smart/batch.pl (accessed on 21 November 2020)) [69]. The putative interacted proteins of ShWRKY41 was predicated by STRING with *Solanum lycopersicum* data [70].

### 4.4. The Expression Analysis of Target WRKYs

The expression levels of *ShWRKY40*, *ShWRKY41*, and *ShWRKY44* under biotrophic pathogens *On*-lz, *Pseudomonas syringae* pv. tomato (*Pst*) DC3000 and nectrophic pathogen *Botrytis cinerea* B05 infections were analyzed using qRT-PCR. *On*-lz was inoculated with the above method, and *Pst* DC3000 and *B. cinerea* B05 were inoculated according to Iwaseer et al. [71] and Lian et al. [72], respectively. All the samples were collected at 0 h, 12 h, 24 h, 36 h, 48 h, 72 h, 96 h, and 120 h post inoculation. Total RNA was extracted according to the above descriptions. Complementary DNA (cDNA) synthesis was performed using a PrimeScript™ RT Reagent Kit with gDNA Eraser (Takara Biotechnology Co., Ltd., Dalian, China) according to the manufacturer’ s instructions and the generation was diluted 10-fold. DNA primers for qRT-PCR (Supplementary Table S2) were designed using Beacon Designer 7.7 (Premier Biosoft, Palo Alto, CA, USA). PCR reaction components and cycling parameters were same with our former description [60]. mRNA expression values were calculated using the 2^–ΔΔCT^ method [73], using *GLYCERALDEHYDE-3-PHOSPHATE DEHYDROGENASE* (*SlGAPDH*) as an internal control.

### 4.5. The Promoter Analysis of ShWRKY41

Firstly, the promoter of *ShWRKY41* (P*_ShWRKY41_*) (0_to_−2000) was analyzed by PlantCARE and Softberry. Next, the promoter of *ShWRKY41* was cloned into the expression vector pCAMBIA0390-GUS using the DNA primers P*_ShWRKY41_*-F (5′-tggctgcaggtcgacggatccCCCTCAACCTATGTCCGAAATC-3′) and P*_ShWRKY41_*-R (5′-tcttagaattcccggggatccGGCTATACCCTTCACCCTCTG-3′), and the recombinant vector was transformed into *Agrobacterium* strain GV3101 for transient expression [74]. *Agrobacterium*-mediated transient assay was performed on the 4-week-old *N. benthamiana* leaves, according to the former method. In brief, the *N. benthamiana* for transient expression were cultivated in a 22 °C chamber with 16 h light/8 h dark cycle for 2 days before the treatment with 100 mM MeJA, 10 mM SA, 0.5 mM ACC (Sigma, Shanghai, China) (1-aminocyclopropanecarboxylic acid, the ethylene precursor and after application, ACC is immediately converted to ethylene by ACC oxidases (ACOs) in plant cells [75]), and water (Control), respectively. All treatments were three replicates, each of which contained three seedlings. After 48 h treatments, the tobacco leaves were collected for detection of GUS activity. Histochemical GUS assay was performed according to the procedure of Jefferson [76].

### 4.6. Expression Analysis of ShWRKY41 in Wild Tomato LA1777 under Abiotic Stresses

For the evaluation of *ShWRKY41* mRNA accumulation under abiotic stresses, 2-week-old wild tomato LA1777 seedlings were used. The samples were collected at 0, 1, 3, 6, and 10 h post treatment, including heat (40 °C), chilling (8 °C), 1 mM SA (salicylic acid), 100 μM MeJA (methyl jasmonate), and 0.5 mM ACC (1-aminocyclopropanecarboxylic acid, the ethylene precursor). Total RNA, cDNA, and qRT-PCR analysis were same with the above descriptions. DNA primers for qRT-PCR (Supplementary Table S2) were designed using Beacon Designer 7.7.

### 4.7. TRV Vectors Construction and Plant Transformation

Virus-induced gene silencing (VIGS) vectors were constructed using tobacco rattle virus (TRV1 and TRV2). The region of target gene for genome-wide off-target gene silencing was selected using SGN VIGS Tool [77]. A 257 bp fragment of *ShWRKY41,* containing a *BamH*I restriction enzyme site, was amplified from the wild tomato LA1777 cDNA and cloned into the expression vector pTRV2 via forward primer (5′- agaaggcctccatggggatccATCCCTAAAGCAACTTCACTTG-3′) and reverse primer (5′- cgtgagctcggtaccggatccATTCCTCAGGAGAAGCTAATGG-3′) according to the method of Senthil-Kumar et al. [78]. After verification using Sanger sequence, the recombinant plasmid was extracted using the E.Z.N.A. Plasmid DNA Mini Kit (Omega Bio-tek, Inc, Doraville, GA) and transformed into *A. tumefaciens* strain GV3101 using the heat shock method [79]. *A. tumefaciens* srtain GV3101 carrying pTRV2 or pTRV2 derivatives, were cultured and infiltrated as previously described [9,60]. Following inoculation, seedlings were transferred to an environmentally controlled growth chamber (25 °C, 16-h light/8-h dark photoperiod). *A. tumefaciens* strain GV3101 carrying pTRV2-*PDS*, constructed before, was used in this study. Photobleaching symptoms in the *PDS*-silenced seedlings were observed at approximately 35 days after inoculation.

### 4.8. Subcellular Localization Analysis

The full-length cDNA of *ShWRKY41* was cloned into the binary vector pBIN-*EGFP* (harboring GFP label) via *BamH* I restriction enzyme digestion followed by ligation of gene-specific DNA primers (Supplementary Table S2). The resultant expression plasmid was transformed into *A. tumefaciens* strain GV3101. *A. tumefaciens* harboring pBIN-*ShWRKY41* was cultured and centrifuged according to the former report [9,60]. Leaves of *N. benthamiana* were inoculated with strains containing recombinant pBIN-*ShWRKY41* or the empty vector pBIN-*EGFP*. GFP fluorescence was detected using a FV1000 confocal microscope (Olympus GmbH, Hamburg, Germany) equipped with a 488 nm filter. The experiment was repeated three times.

### 4.9. Fungal Biomass Analysis and Quantification of Disease Severity

For each experiment, two subsets of plants were maintained from each treatment (i.e., TRV2, TRV2-*SlPDS*, or TRV2-*ShWRKY41*). After 35 days inoculation, samples were collected from TRV2, TRV2-*SlPDS,* or TRV2-*ShWRKY41* silenced seedlings. Total RNA, synthesis of cDNA was performed according to the above descriptions. Silencing efficiency was evaluated by qRT-PCR using gene-specific primers (Supplementary Table S2). In parallel to sample for mRNA analysis, samples were collected at 3-time points (12, 36, and 72 hpi) for histological observation. Disease severity was assessed by the former description with 0–9 disease rating scale [80]: 0 = no disease symptoms; 1 = 0–5% of leaves having disease symptoms; 3 = leaves with infection lesions comprising up to 6–10% of the total leaf surface; 5 = leaves with infection lesions up to 11–20% of the total leaf surface; 7 = leaves with infection lesions up to 21–40% of the total leaf surface; and 9 = leaves with infection lesions up to 41–100% of the total leaf surface.

Disease severity indices were calculated using the following equation: Disease index (DI) = [Σ (number of diseased plant leaves at a given disease severity × the disease severity)/(total plant leaves analyzed × 9)] × 100. An average DI was calculated at three independent time points for each infected plant.

To quantify the accumulation of H_2_O_2_ (H_2_O_2_ production rate = H_2_O_2_ numbers per 100 penetration site) and the induction of HR cell death (HR production rate = HR numbers per 100 penetration site) during *On*-lz infection, the 3,3-diaminobenzidine (DAB; AMERCO, Solon, OH, USA) and trypan blue staining methods were used, respectively [61,81]. In brief, samples collected from 5-time points (12, 36, and 72 hpi) were cut into 2–3 cm^2^ segments without the edge and main vein, and then stained as previous description [61,81]. In addition, the samples, staining with DAB, were re-staining with 5% Coomassie brilliant blue G-250 for infection structures observation. At least 30 penetration sites on each of four-leaf samples were observed at each time point. Statistical significance was assessed by a Student’s *t*-test (α = 0.05.) using the SPSS software 20.0 (IBM, New York, NY, USA).

### 4.10. The expression Analysis of Plant Hormone Biosynthesis Related Genes

To know which hormone involved in wild tomato *S. habrochaites* LA1777 against *On*-lz, based on the result of *ShWRKY41* expression analysis and GUS-promoter assay, *PLA4*, *ICS*, *ACS3*, and *ACO2* were chosen to monitor the hormone biosynthesis process. *PLA4* and *ICS*, coding protein and conferring 10% and 90% SA biosynthesis in plant [82,83], are the key genes in SA biosynthesis. And the products of *ACS3* and *ACO2* are the key enzymes of Eth biosynthesis [84,85,86]. The samples were collected from wild tomato LA1777 and cultivar tomato MM after *On*-lz inoculation 0, 12, 36, 48, 72, 96, and 120 h. And total RNA, cDNA and qRT-PCR were carried out according to the above description.

### 4.11. Data Collection and Analysis

All experiments were performed in triplicate and at least 30 penetration sites were scored by microscopy at each time point. Statistical analyses were carried out using the IBM SPSS statistics software package (version 20.0). Comparisons between control samples and each treatment were evaluated using a Student’s *t*-test at a significance level of α = 0.05.

## Figures and Tables

**Table 1:** Categorization of differentially expressed *ShWRKYs* under *On*-lz infection in wild tomato LA1777.

	Gene_Description	Group	log2FoldChange	padj
12 hpi	*ShWRKY44*	I	4.99	0.000017
	*ShWRKY1*	I	2.58	0.000000
	*ShWRKY40*	II-a	2.57	0.000442
	*ShWRKY51*	II-c	−1.83	0.000173
	*ShWRKY IId-1*	II-d	1.46	0.010183
	*ShWRKY22-like*	II-e	1.90	0.021111
	*ShWRKY29*	II-e	1.64	0.000160
	*ShWRKY41*	III	2.37	0.000000
36 hpi	*ShWRKY44*	I	5.73	0.007329
	*ShWRKY33B*	I	2.71	0.000026
	*ShWRKY1*	I	1.66	0.003228
	*ShWRKY3*	I	−1.33	0.047367
	*ShWRKY45*	II-a	4.45	0.011781
	*ShWRKY40*	II-a	2.76	0.000469
	*ShWRKY9*	II-b	5.18	0.029976
	*ShWRKY30*	II-c	2.72	0.009789
	*ShWRKY30-like*	II-c	1.48	0.029717
	*ShWRKY IId-1*	II-d	1.51	0.013714
	*ShWRKY22-like*	II-e	2.06	0.004722
	*ShWRKY26*	II-e	1.75	0.029760
	*ShWRKY53*	III	2.49	0.000276
	*ShWRKY53*	III	2.26	0.000432
	*ShWRKY41*	III	2.10	0.000002
72 hpi	*ShWRKY33B*	I	3.13	0.000000
	*ShWRKY1*	I	3.04	0.000000
	*ShWRKY31*	I	1.76	0.000000
	*ShWRKY14*	I	−2.69	0.038904
	*ShWRKY40*	II-a	4.54	0.000000
	*ShWRKY48*	II-c	2.28	0.000019
	*ShWRKY23*	II-c	1.68	0.001020
	*ShWRKY56*	II-c	5.45	0.009433
	*ShWRKY75*	II-c	−1.57	0.048565
	*ShWRKY IId-1*	II-d	2.28	0.000000
	*ShWRKY7*	II-d	1.44	0.000000
	*ShWRKYIId-4*	II-d	1.20	0.003540
	*ShWRKY21*	II-d	−1.14	0.025213
	*ShWRKY22-like*	II-e	3.26	0.000000
	*ShWRKY26*	II-e	3.12	0.000000
	*ShWRKY70*	II-e	1.55	0.000000
	*ShWRKY41*	III	3.06	0.000000
	*ShWRKY53*	III	3.00	0.000000
	*ShWRKY53*	III	2.11	0.000000

Note: padj is the adjusted *p* value using the Benjamini and Hochberg’s approach.

## Data Availability

Data supporting the reported results can be found in the supplementary materials file.

## Supplementary Materials

The following are available online at https://www.mdpi.com/article/10.3390/ijms23031267/s1.
