# Supplementary material for: Identification and Characterization of WRKY41, a Gene Conferring Resistance to Powdery Mildew in Wild Tomato (Solanum habrochaites) LA1777
Source: Int J Mol Sci. 2022 Jan 23;23(3):1267. doi: 10.3390/ijms23031267 (PMC8836203; doi:10.3390/ijms23031267)
Supplement: Supplementary file 1 [file ijms-23-01267-s001.zip › ijms-1529056-supplementary figure.pdf]

Supplementary materials

A B

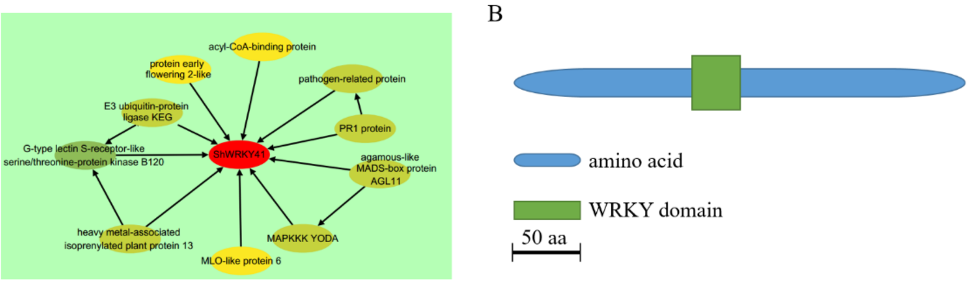

C

Gene_id	T2vsCK2_log2FoldChange	T2vsCK2_padj	T3vsCK3_log2FoldChange	T3vsCK3_padj	T4vsCK4_log2FoldChange	T4vsCK4_padj	Gene_description
100191111	-0.96145	0.065152	-1.97558	0.051175	0.870821	0.032153	PR1 protein
101265726	-1.99215	0.07529	-1.10398	0.178298	-0.45176	0.530061	pathogen-related protein
101245171	-0.99768	0.19249	-1.28453	0.139899	-0.17412	0.777812	MLO-like protein 6
101263615	0.148196	0.751265	-0.40737	0.394906	-0.00251	0.993848	mitogen-activated protein kinase kinase kinase YODA

Figure S1. The function analysis of ShWRKY41. (**A**) The interaction proteins of ShWRKY41 in *Solanum lycopersicum* by STRING v11.5, and the figure was performed with Cytoscope_v3.8.1. (**B**) The structure of ShWRKY41analyzed by Pfam, in which the bar means 50 amino acid. (**C**) The expression level of genes, coding the proteins interacting with ShWRKY41, in RNA-seq data.
